# Resolution of Symptoms of Suspected Nonatypical Endometrial Hyperplasia Using Herbal Medicine Modified Sihogyeji-Tang Monotherapy: A Case Report with Ultrasound Monitoring

**DOI:** 10.3390/life15020256

**Published:** 2025-02-07

**Authors:** Eunbyul Cho, Pyung-Wha Kim, Cheol-Hyun Kim, Changsop Yang, Stella Roh

**Affiliations:** 1 KM Science Research Division, Korea Institute of Oriental Medicine, Daejeon 34054, Republic of Korea; eunbc@kiom.re.kr (E.C.); peace35@kiom.re.kr (P.-W.K.); yangunja@kiom.re.kr (C.Y.); 2 Department of Internal Medicine, College of Korean Medicine, Wonkwang University, Iksan 54538, Republic of Korea; user2307@hanmail.net; 3 Kirin Korean Medicine Clinic, Incheon 21574, Republic of Korea

**Keywords:** endometrial hyperplasia, hypomenorrhea, Chai-hu-gui-zhi-tang, Saiko-keishi-to, ultrasonography

## Abstract

This case report presents the therapeutic effects of herbal medicine modified Sihogyeji-tang monotherapy for suspected nonatypical endometrial hyperplasia (EH), documented through transabdominal ultrasonography monitoring and clinical symptom assessment. A 38-year-old woman presented with hypomenorrhea and ovulation-related pain, headache, body aches, and nausea since April 2023. The patient was clinically assessed as having nonatypical EH based on the ultrasound findings and clinical symptoms. She was treated with modified Sihogyeji-tang twice daily from 3 June 2023 to 29 December 2023. Treatment outcomes were evaluated using regular transabdominal ultrasonography measurements of endometrial thickness and changes in menstrual patterns, including cycle length and blood volume. Menstrual symptoms showed notable improvements: severe ovulation pain decreased from NRS 7–8 to 0, menstrual volume increased from 2 to 3 medium pads to 4 to 5 large pads per day during peak flow, and menstrual duration normalized from 4 to 6 days. Symptoms associated with ovulation and menstruation, headache, chills, and nausea resolved. At the 3-month follow-up visit after 7 months of herbal medicine treatment, endometrial thickness measured during the secretory phase had normalized to 1.40 cm (normal range: 0.7–1.4 cm). The coexisting uterine myoma remained stable throughout the treatment and follow-up. No adverse events were reported during the entire course of treatment. This case demonstrated that modified Sihogyeji-tang alone may effectively improve suspected nonatypical EH and its associated symptoms. Improvement was objectively assessed using ultrasound measurements and was sustained over a 9-month follow-up.

## 1. Introduction

Endometrial hyperplasia (EH) is an abnormal proliferation of the endometrium resulting from continuous estrogen stimulation in the absence of sufficient progesterone opposition [1,2]. It presents with symptoms such as abnormal menstrual bleeding or lower abdominal pain, requiring appropriate treatment for symptom improvement and endometrial normalization [3]. Initial diagnosis typically involves transvaginal ultrasonography screening, followed by endometrial sampling and histological examination when indicated [3]. Through this diagnostic process, EH is classified into nonatypical and atypical types. Atypical EH is considered a precursor lesion that can progress to carcinoma and primarily requires surgical treatment.

Most nonatypical cases can be managed with hormone therapy or curettage [4], but conventional treatment methods have several limitations. Hormone (progestin) therapy can cause side effects such as weight gain, nausea, irregular vaginal bleeding, and venous thromboembolism [4]. While dilatation and curettage provide both diagnostic and therapeutic value, they are invasive procedures with risks of complications and potential recurrence. Additionally, hysterectomy provides a definitive treatment but eliminates future fertility potential and may be overly aggressive for nonatypical cases. Furthermore, patient compliance with long-term hormonal therapy can be challenging, and some patients may seek less invasive alternatives [5]. Evidence for current hormonal treatments of EH is primarily derived from case studies, and the lack of standard and low-risk treatment options highlights the importance of exploring alternative therapeutic approaches [2].

For EH, herbal medicine offers a conservative and safe alternative to conventional treatments such as hormone therapy, curettage, or hysterectomy. According to a systematic review of Chinese clinical studies, herbal medicine showed significantly higher effect rates than oral hormone therapy (risk ratio = 1.22, 95% confidence interval = 1.10–1.35, *p* = 0.0002) [6]. Previous case studies using Guichulpajing-tang and Eunhwasagan-tang for EH reported improvements in endometrial thickness and clinical symptoms such as abnormal bleeding and pain [7,8]. However, these case studies involved complex Korean Medicine treatments, such as acupuncture and pharmacopuncture, alongside herbal medicine, making it difficult to assess the therapeutic effect of herbal medicine alone. To date, clinical research regarding EH in traditional medicine is limited.

Sihogyeji-tang (Chai-hu-gui-zhi-tang in Chinese, Saiko-keishi-to in Japanese), a traditional herbal formula combining Soshiho-tang (Xiao-chai-hu-tang) and Gyeji-tang (Gui-zhi-tang), has been traditionally used for conditions presenting with both exterior and interior symptoms, such as chilling and digestive discomfort [9,10]. While effective herbal medicine treatments for EH remain limited in the literature, we report a case where modified Sihogyeji-tang, prescribed based on the patient’s systemic symptoms, examination, and pattern identification, resulted in notable improvement of symptoms and reduction in endometrial thickness. Unlike previous case studies that combined various treatments with herbal medicines [7,8], the present study specifically focused on the therapeutic effects of herbal medicine monotherapy. Furthermore, we provided objective evidence of treatment outcomes through quantitative measurements of endometrial thickness using abdominal ultrasound imaging.

## 2. Case Presentation

A 38-year-old Korean woman presented with ovulation-related symptoms and hypomenorrhea. The patient was unmarried and had no history of pregnancy, childbirth, or other medical history. Her menstrual cycle was regular, at approximately 31 days. She worked in an office. Despite experiencing chronic indigestion, palpitations, and anxiety with coffee consumption, she consumed three to four cups of coffee daily. The patient’s height was 156 cm, weight was 57 kg, and BMI was 19.3 kg/m^2^, indicating a normal weight range.

Since April 2023, during ovulation, the patient has experienced severe headaches and body aches for two days, accompanied by brown-colored vaginal discharge and severe nausea, requiring approximately two painkillers per day. Her menstrual period shortened from the usual 6 to 4 days, and headaches and chills became more severe than the menstrual cramps. The patient reported experiencing significant stress at the time of the onset. She did not visit other hospitals for examination or treatment of symptoms and first visited a Korean Medicine clinic on 3 June 2023 (day 22 of her menstrual cycle). Her chief complaints were severe ovulation pain (NRS, 7–8) with headaches and nausea, headaches, and chills during menstruation, and decreased menstrual flow. Abdominal examination revealed that the chest and hypochondrium were bloated and distressed. Pulse diagnosis revealed string-like, tight left and right cubit pulses.

For diagnostic evaluation, transabdominal ultrasonography was performed by a doctor of Korean Medicine certified as a Registered Diagnostic Medical Sonographer in Obstetrics and Gynecology by the American Registry for Diagnostic Medical Sonography, with over 20 years of experience in ultrasonography. Ultrasound examinations were performed using a GE Healthcare LOGIQ P9 with a C1-5 Convex probe at a frequency of 4 MHz. The transducer was positioned on the lower abdomen cephalic to the pubic bone, and the uterus was identified by scanning longitudinally parallel to the suprapubic midline, followed by image optimization. Endometrial thickness was measured and interpreted in relation to the menstrual cycle phase. During the examination, a suspected uterine myoma was detected, and its size was evaluated by measuring the maximum distances of the margins in the transverse and longitudinal directions.

At the initial visit (day 22 of the first menstrual cycle), the endometrial thickness was 2.1 cm, and a uterine myoma measuring 1.42 cm × 1.10 cm was also noted. Based on the abdominal ultrasound findings showing relatively homogeneous endometrial echogenicity, simple hyperplasia was inferred. Endometrial curettage and biopsy could not be performed at a Korean Medicine clinic. Although referral to a secondary hospital was recommended for definitive diagnosis through endometrial biopsy, the patient opted to begin with herbal medicine treatment to address her immediate symptoms. As an unmarried woman planning a future pregnancy, the patient was hesitant to undergo surgical intervention or hormone therapy because of concerns regarding their potential impact on fertility.

Based on the presentation of both exterior (headache, chills) and interior symptoms (nausea, chronic indigestion), along with abdominal examination findings, including chest and hypochondrial bloating and distress, the patient’s condition was diagnosed as a ‘half-exterior and half-interior pattern,’ and a modified Sihogyeji-tang was prescribed. The herbal decoction was administered twice daily at 120 mL per dose (Table 1). Treatment was provided for approximately seven months, from the initial visit on 3 June 2023 to 29 December 2023. No other Korean Medicine treatments, such as acupuncture or moxibustion, were administered. As the patient consumed 3–4 cups of coffee daily, she was advised to minimize caffeine intake. The patient reduced her coffee intake to one cup per day during the first month and discontinued it from the second month. Additionally, she was instructed to perform pelvic stretching exercises twice daily, in the morning and evening.

After two weeks of herbal medicine treatment (visit 2, day 7 of the 2nd menstrual cycle), the patient’s menstrual volume, assessed by the average number of pads used on days 1 and 2 of menstruation, was increased to 4 to 5 medium pads per day. Although it would be ideal to assess the entire menstrual period, menstrual volume was measured specifically on days 1 and 2, as day 2 generally had the heaviest flow, making it easier to assess in a clinical setting [11]. After five weeks of herbal medicine treatment (visit 3, day 3 of the 3rd menstrual cycle), she reported that ovulation pain and headache decreased to NRS 1–2, eliminating the need for analgesics. The patient was menstruating at the time of the visit and reported no headache or chills.

Three months after taking the herbal medicine (visit 4, day 19 of the 4th menstrual cycle), menstrual volume further increased to 4 to 5 large pads per day on menstrual days 1 and 2, returning to her previous menstrual volume with menstrual pain at NRS 3. Ovulation-related symptoms, including pain, headache, body aches, nausea, and bleeding, resolved. On 30 October 2023 (visit 5, day 11 of the 6th menstrual cycle), her menstrual period returned to 6 days. Increased menstrual volume remained at 4–5 large pads per day. After approximately seven months of herbal medicine treatment, by 29 December 2023 (visit 6, day 10 of the 8th menstrual cycle), menstrual pain had almost completely resolved to an NRS score of 0–1. Given the significant improvement in ovulation-related symptoms and menstrual pain, herbal medicine was discontinued after this visit.

**Figure 1:**
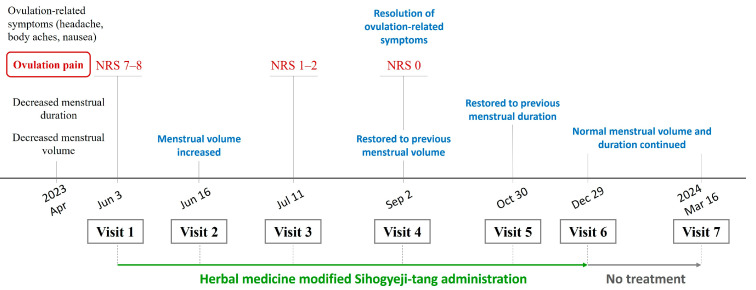
Timeline of major clinical events and treatment course.

**Figure 2:**
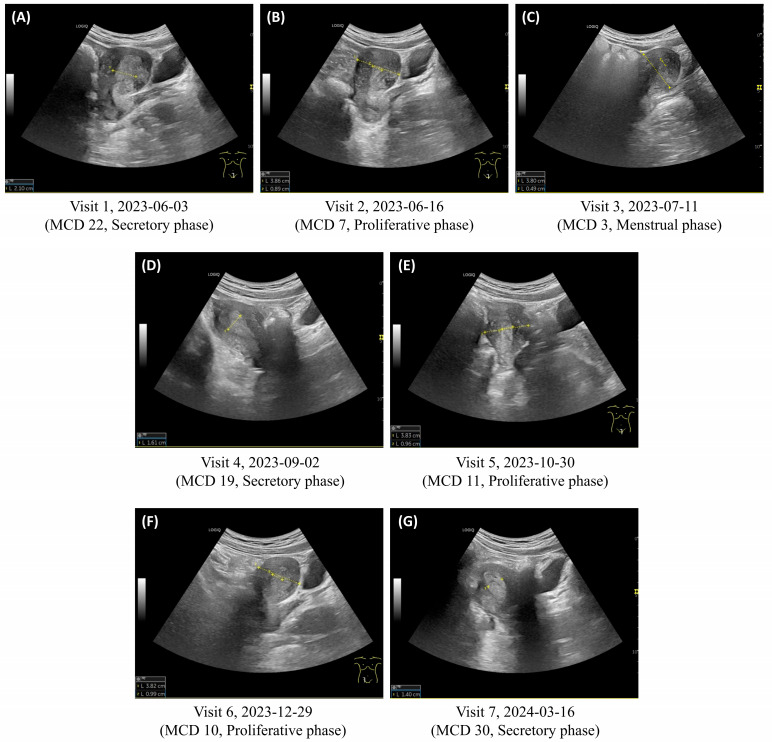
Transabdominal ultrasound images showing endometrial thickness measurements. Normal ranges by menstrual cycle phase are proliferative phase (0.4–0.8 cm), secretory phase (0.7–1.4 cm), and menstrual phase (0.1–0.4 cm). (**A**) Visit 1: endometrium measuring 2.10 cm in the secretory phase, exceeding the normal range. (**B**) Visit 2: 0.89 cm in the proliferative phase, exceeding the normal range. (**C**) Visit 3: 0.49 cm during the menstrual phase, exceeding the normal range. (**D**) Visit 4: 1.61 cm in the secretory phase, exceeding the normal range. (**E**) Visit 5: 0.96 cm in the proliferative phase, exceeding the normal range. (**F**) Visit 6: 0.99 cm in the proliferative phase, exceeding the normal range. (**G**) Visit 7: 1.40 cm in the secretory phase, within the normal range. MCD, menstrual cycle day.

At the final follow-up visit on 16 March 2024 (visit 7, day 30 of the 10th menstrual cycle), menstrual pain remained at NRS 0–1, and menstrual volume was maintained at 4–5 large pads per day on days 1 and 2 of menstruation (Figure 1, Table 2). Endometrial thickness, which consistently exceeded the normal range at previous visits, normalized within the reference range (1.40 cm) at the final visit (Figure 2). The uterine myoma, initially measured at 1.42 cm × 1.10 cm, remained stable throughout the treatment period (Supplementary Figure S1). No adverse events or unexpected incidents occurred during the treatment. This case report was approved by the Institutional Review Board of Wonkwang University (WKUIRB 202410-103-01).

### Patient Perspective

The patient demonstrated high adherence to herbal medicine treatment, which was reflected in consistent medication intake and regular follow-up visits for monitoring therapeutic efficacy. At the end of treatment, the patient stated, “As an unmarried woman, I had concerns about my future fertility, but I am now pleased that all my symptoms have improved with herbal medicine without surgery”.

## 3. Discussion

The incidence of EH has been increasing recently with the rising prevalence of its risk factors, including polycystic ovary syndrome and obesity [3]. Among the types of EH, atypical endometrial intraepithelial neoplasia is considered a precursor lesion to endometrial cancer. It requires aggressive intervention, such as surgery, while all other benign variants, such as nonatypical EH, are primarily managed through noninvasive medical treatment [4]. Korean Medicine treatment for EH mainly focuses on simple nonatypical hyperplasia [3]. Regular monitoring of endometrial thickness through imaging findings is essential to assess treatment response and ensure appropriate management of EH. In this case, we measured endometrial thickness using transabdominal ultrasonography at each visit and assessed the patient’s condition by comparing measurements with cycle-specific reference ranges (proliferative phase: 0.4–0.8 cm, secretory phase: 0.7–1.4 cm, menstrual phase: 0.1–0.4 cm) [12].

Sihogyeji-tang, one of the 56 Korean Medicine prescriptions covered by national health insurance, is composed of Bupleuri Radix, Cinnamomi Ramulus, Scutellariae Radix, Ginseng Radix, Paeoniae Radix, Pinelliae Tuber, Glycyrrhizae Radix et Rhizoma, Zingiberis Rhizoma, and Zizyphi Fructus [10]. In this case, as the patient presented with both exterior (headache, chills) and interior symptoms (nausea, chronic indigestion), we used Sihogyeji-tang as the base formula, which is indicated for the ‘half-exterior and half-interior pattern’. Sihogyeji-tang has been reported to have protective effects against gastric mucosal damage [13], and there are case reports of its use in nonconvulsive status epilepticus with delirium [14] and allergic dermatitis [9]. However, clinical reports regarding menstrual disorders are scarce, indicating the need for further research. Rehmanniae Radix Recens was used as the chief herb because of its dual action of nourishing blood and generating fluids, and it is traditionally used for hemorrhagic disorders such as metrorrhagia [15]. Furthermore, Rehmanniae Radix Recens has been reported to be effective for various inflammatory and metabolic diseases [16] and to induce apoptosis in cervical carcinoma [17], and is presumed to have contributed to the improvement of EH symptoms in this case.

The normalization of endometrial thickness observed in this case suggests that herbal medicines can inhibit abnormal endometrial proliferation. It is particularly noteworthy that the endometrial thickness decreased to within the normal range without hormone therapy, such as progesterone. The normalization of menstrual volume and improvement in menstrual and ovulation pain demonstrate that herbal medicine treatment not only reduces endometrial thickness but also improves overall menstrual physiology. The improvement in systemic symptoms, such as headaches and chills, demonstrated that herbal medicine can effectively treat both local endometrial lesions and systemic symptoms.

In South Korea, primary Korean Medicine clinics have limited capacity to perform endometrial biopsies, requiring patient referral to medical centers for definitive diagnosis. In this case, while referral for biopsy was recommended, the patient primarily sought symptom relief, leading to our decision to initiate herbal medicine treatment based on Korean Medicine diagnosis and abdominal ultrasound findings. Although the symptoms showed significant improvement with herbal medicine monotherapy, we consistently emphasized to the patient the importance of obtaining a definitive diagnosis through biopsy at a secondary hospital. While the lack of histological confirmation through biopsy represents a limitation of this case, our findings demonstrate that herbal medicine can effectively alleviate symptoms in the primary care management of suspected endometrial hyperplasia, offering insights into conservative treatment options for patients who initially prefer noninvasive approaches.

The main limitations of this case report include its nature as a single case and the potential confounding effects of lifestyle changes, particularly reduced caffeine intake, which makes it challenging to isolate the specific effects of modified Sihogyeji-tang. The effects of coffee on menstruation or the endometrium are controversial. It is suggested that caffeine should be considered a risk factor for most menstrual abnormalities [18]. On the other hand, caffeine consumption was not associated with premenstrual syndrome in a prospective study. A meta-analysis revealed the association of coffee consumption with reduction in risk of endometrial cancer [19,20]. Although the effect of pelvic stretching on EH has not been well studied, considering the previous result that stretching reduced pain in girls with primary dysmenorrhea [21], it might have contributed to relieve the patient’s menstrual pain.

Nevertheless, this case demonstrates meaningful clinical improvements in a patient with suspected EH using herbal medicine monotherapy without progesterone. The treatment resulted in both objective improvements, evidenced by normalized endometrial thickness on ultrasound, and comprehensive symptom relief, including resolution of ovulation-related symptoms and normalization of menstrual patterns. The therapeutic effects were sustained through the three-month post-treatment follow-up period. While these results are promising, further case series and large-scale clinical studies are needed to validate the therapeutic effects of herbal medicines for endometrial hyperplasia and other estrogen-dependent conditions.

## 4. Conclusions

This case demonstrates the efficacy of modified Sihogyeji-tang monotherapy in a patient with suspected EH, as examined using ultrasonography. Through herbal medicine treatment, clinical symptoms, including decreased menstrual flow, ovulation pain, and headache, also improved, with therapeutic effects sustained throughout the nine-month follow-up.

## Figures and Tables

**Table 1:** Composition of modified Sihogyeji-tang.

Scientific Name	Herbal Name	Chinese Name	One-Day Dose (g)
*Rehmannia glutinosa* Liboschitz ex Steudel	Rehmanniae Radix Recens	生地黃	8.0
*Angelica gigas* Nakai	Angelicae Gigantis Radix	當歸	5.2
*Bupleurum falcatum* Linné	Bupleuri Radix	柴胡	5.2
*Poria cocos* Wolf	Poria Sclerotium	茯苓	5.2
*Paeonia suffruticosa* Andrews	Moutan Cortex	牧丹皮	2.6
*Salvia miltiorrhiza* Bunge	Salviae Miltiorrhizae Radix	丹蔘	2.6
*Alisma orientale* Juzepczuk	Alismatis Rhizoma	澤瀉	2.6
*Glycyrrhiza uralensis* Fischer	Glycyrrhizae Radix et Rhizoma	甘草	2.6
*Scutellaria baicalensis* Georgi	Scutellariae Radix	黃芩	2.0
*Panax ginseng* C.A. Meyer	Ginseng Radix	人蔘	2.0
*Cinnamomum cassia* Presl	Cinnamomi Ramulus	桂枝	2.0
*Paeonia lactiflora* Pallas	Paeoniae Radix	芍藥	2.0
*Zingiber officinale* Roscoe	Zingiberis Rhizoma Recens	生薑	2.0
*Cnidium officinale* Makino	Cnidii Rhizoma	川芎	2.0
*Zizyphus jujuba* Miller var. inermis Rehder	Zizyphi Fructus	大棗	2.0
*Pinellia ternata* Breitenbach	Pinelliae Tuber	半夏	1.6
*Zingiber officinale* Roscoe	Zingiberis Rhizoma	乾薑	1.2
*Achyranthes bidentata* Blume	Achyranthis Radix	牛膝	1.2
*Carthamus tinctorius* Linné	Carthami Flos	紅花	0.6

**Table 2:** Detailed clinical measurements and observations at each visit.

Visits	Visit Date	Last Menstrual Period	MCD	Menstrual Pain (NRS)	Menstrual Volume * (Pads/Day)	Days of Menstrual Bleeding (Days)	Changes in Main Symptoms
1	3 June 2023	13 May 2023	22	4	2–3 medium pads	4	Ovulation-related symptoms Multiple systemic symptoms (headache, body aches, nausea) Brown discharge Ovulation pain (NRS 7–8) Required analgesics: 3/day × 2 days Headache and chills during menstruation Menstrual duration decreased from 6 to 4 days
2	16 June 2023	10 June 2023	7	3	4–5 medium pads	4	Increased menstrual volume
3	11 July 2023	9 July 2023	3	3	4–5 medium pads	3 ^#^	Decreased ovulation pain, headache (NRS 1–2) (discontinued analgesics) No headache or chills during menstruation
4	2 September 2023	15 August 2023	19	3	4–5 large pads	4	Resolution of ovulation-related symptoms (pain, headache, body aches, nausea, bleeding) Restored to previous menstrual volume
5	30 October 2023	20 October 2023	11	3	4–5 large pads	6	Restored to previous menstrual duration
6	29 December 2023	20 December 2023	10	0–1	5–6 large pads	6	Almost no menstrual pain, normal menstrual volume
	* Herbal medicine treatment completed						
7	16 March 2024	17 February 2024	30	0–1	4–5 large pads	6	Continued absence of ovulation-related symptoms, normal menstrual volume

MCD, menstrual cycle day. NRS, numeric rating scale. * Number of pads used per day on menstrual days 1 and 2. ^#^ Patient was on day 3 of menstruation at the time of visit; total duration could not be determined.

## Data Availability

The original contributions presented in this study are included in the article and Supplementary Material. Further inquiries can be directed to the corresponding author.

## Supplementary Materials

The following Supporting Information can be downloaded at: https://www.mdpi.com/article/10.3390/life15020256/s1, Figure S1: The patient’s transabdominal ultrasound images showing size of suspected myoma.
