# Supplementary material for: Resolution of Symptoms of Suspected Nonatypical Endometrial Hyperplasia Using Herbal Medicine Modified Sihogyeji-Tang Monotherapy: A Case Report with Ultrasound Monitoring
Source: Life (Basel). 2025 Feb 7;15(2):256. doi: 10.3390/life15020256 (PMC11856214; doi:10.3390/life15020256)
Supplement: Supplementary file 1 [file life-15-00256-s001.zip › life-3462469-supplementary.pdf]

Supplementary Figure S1. The patient's transabdominal ultrasound images showing size of suspected myoma

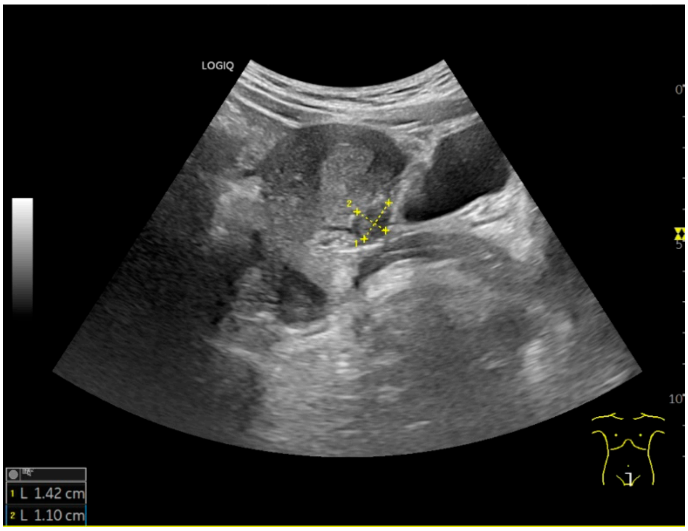

Visit 1, 2023-06-03

Transabdominal ultrasound image showing an intramyometrial mass with dimensions of **1.42cm** × **1.10cm**, suspected myoma.

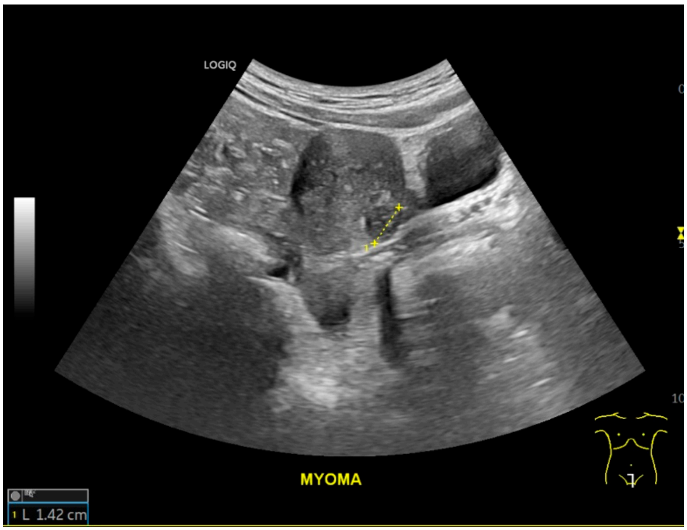

Visit 2, 2023-06-16

Transabdominal ultrasound image showing a suspected myoma with a diameter of **1.42cm**.

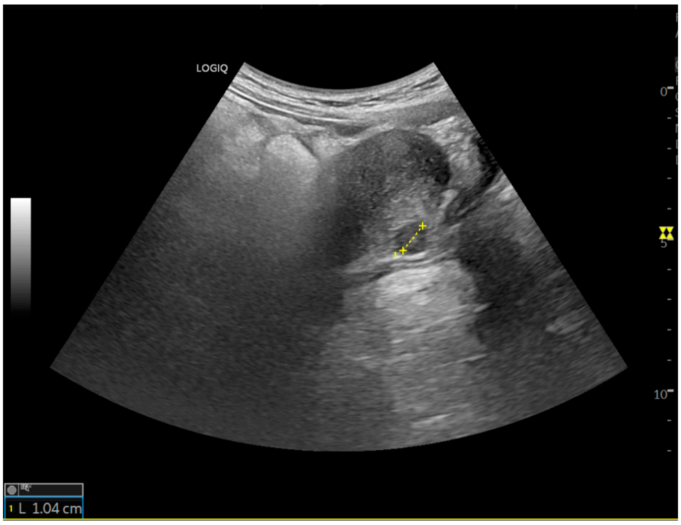

Visit 3, 2023-07-11

Transabdominal ultrasound image showing a suspected myoma with a diameter of **1.04cm**.

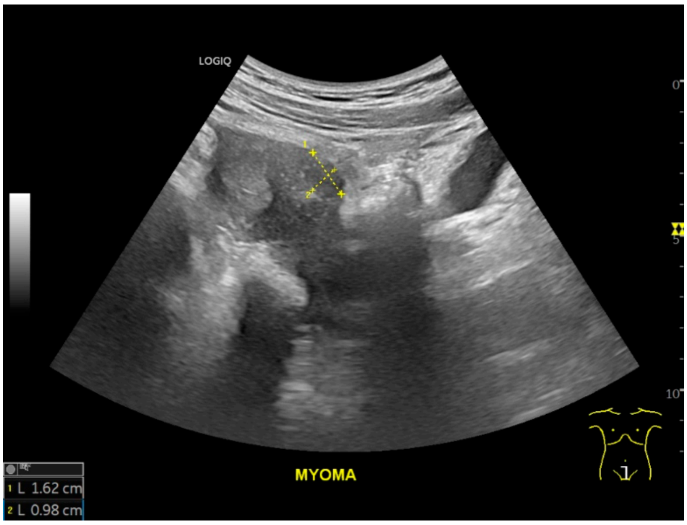

Visit 4, 2023-09-02

Transabdominal ultrasound image showing a suspected myoma with dimensions of **1.62cm** × **0.98cm**.

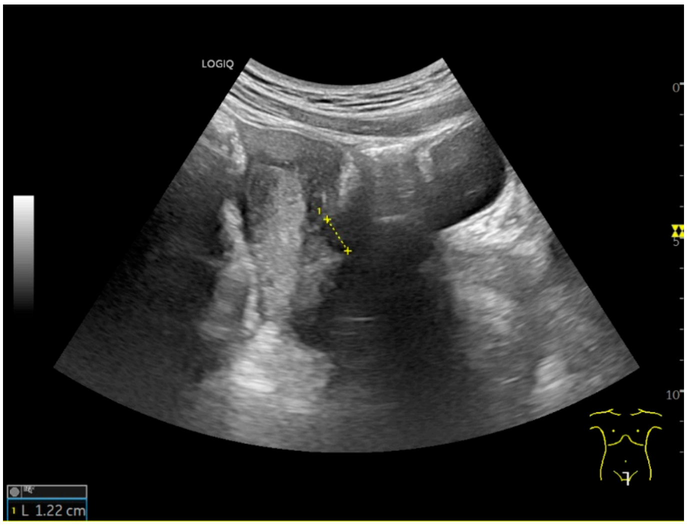

Visit 5, 2023-10-30

Transabdominal ultrasound image showing a suspected myoma with a diameter of **1.22cm**.

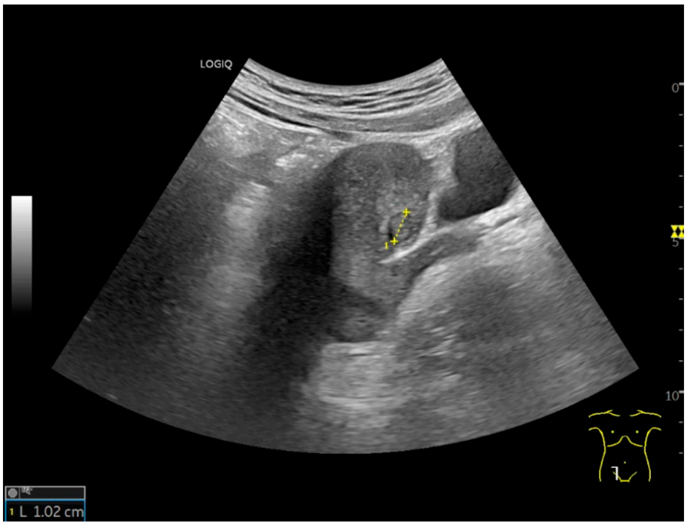

Visit 6, 2023-12-29

Transabdominal ultrasound image showing a suspected myoma with a diameter of **1.02cm**.

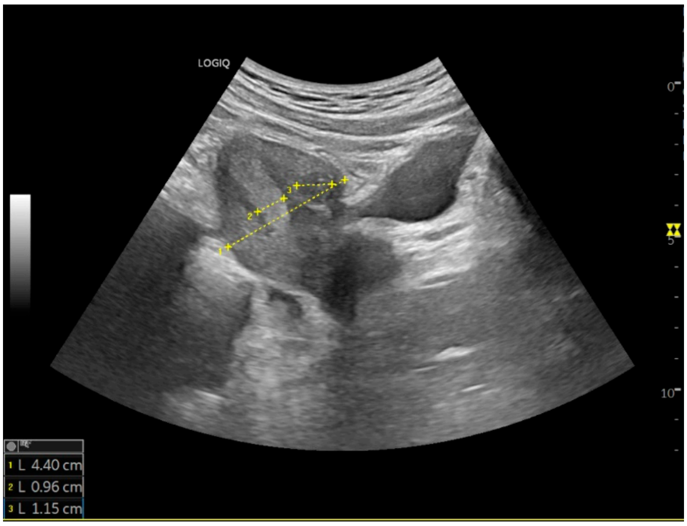

Visit 7, 2024-03-16

Transabdominal ultrasound image showing a suspected myoma with a diameter of **1.15cm**.
